# Hearing loss and cognitive function among Chinese older adults: the role of participation in leisure activities

**DOI:** 10.1186/s12877-020-01615-7

**Published:** 2020-06-19

**Authors:** Jiamin Gao, Nicole M. Armstrong, Jennifer A. Deal, Frank R. Lin, Ping He

**Affiliations:** 1 Guanghua School of Management, Institute of Strategy Research, Peking University, Beijing, 100871 China; 2 Laboratory of Behavioral Neuroscience, National Institute on Aging, National Institutes of Health, Baltimore, MD USA; 3 Department of Epidemiology, Johns Hopkins University Bloomberg School of Public Health, Baltimore, MD USA; 4 Center on Aging and Health, Johns Hopkins University School of Medicine, Baltimore, MD USA; 5 Department of Otolaryngology-Head and Neck Surgery, Johns Hopkins University School of Medicine, Baltimore, MD USA; 6 Department of Mental Health, Johns Hopkins University Bloomberg School of Public Health, Baltimore, MD USA; 7 China Center for Health Development Studies, Peking University, No. 38 Xueyuan Road, Haidian District, Beijing, 100191 People’s Republic of China

**Keywords:** Hearing loss, Activities engagement, Cognitive function, Sex difference

## Abstract

**Background:**

Hearing loss, a highly prevalent sensory impairment affecting older adults, is a risk factor for cognition decline. However, there were very limited studies on this association in low-resource countries. This study aimed to assess the association between self-reported hearing loss and cognitive decline, and whether engagement in leisure activities moderated this association among older adults in China.

**Methods:**

Data were obtained from two waves of the nationally representative survey of China Longitudinal Healthy Longevity Survey (CLHLS) between 2011/12–2014. Eight thousand eight hundred forty-four individuals aged 65 years old or above with a dichotomized measure of self-reported hearing status were included. Modified Mini-Mental Examination (MMSE) was used to measure global cognition. Fixed-effects models were used to estimate whether leisure activity engagement moderated the association of self-perceived hearing loss with global cognitive change in the overall sample and sex subsamples.

**Results:**

Self-reported hearing loss was associated with cognitive impairment, with an odds ratio of 2.48 [1.22, 5.06]. Sex difference in the association of hearing loss and cognitive impairment was not found. Self-reported hearing loss was associated with cognitive decline, with 8% increase in risk compared with those with normal hearing. Frequent engagement in leisure activities moderated the association between hearing loss and cognitive decline for the whole and male samples.

**Conclusion:**

Hearing loss was associated with cognitive decline, and leisure activities engagement moderated the association among males rather than females.

## Background

Dementia is a major global public health concern. It affected approximately 50 million people worldwide [1]. Dementia prevalence is expected to rise in the near future, especially in China, where its prevalence in a population of 1.7 billion adults aged 60 or older [2] was 4.6% (8 million) in 2010 [3], which is expected to increase to 6.7% (23 million people) by 2030 [4]. Cognitive impairment, characterized by the presence of weakening of one or more cognitive domains like immediate and delayed memory, may develop into dementia [5]. The prevalence of mild cognitive impairment among Chinese adults aged ≥60 was 12.7% in 2010 [6]. Cognitive decline is more prevalent in older individuals [7], and previous studies have shown that the presence of chronic diseases, sensory loss, disengagement in physical, mental or social activity, and exposure to acute and chronic stress may be related to poorer cognitive performance in later life [8, 9].

Hearing loss, a prevalent chronic condition in older adults, is a key risk factor of cognitive impairment and dementia [10–12]. Approximately 11% of adults aged ≥60 were diagnosed with disabling hearing impairment in China [13]. Dementia is a major source of disability around the world, and no disease-modifying treatments so far [14]. Age-related hearing loss may be associated with an increased risk of dementia in later life, which has garnered increasing attention as a potentially modifiable risk factors for dementia and cognitive decline [10–12]. Evidence from a randomized controlled trial regarding hearing support in dementia (i.e., SENSE-Cog Field Trial) demonstrated that employing a sensory intervention to support hearing, such as providing hearing aids, communication training, and supplementary sensory aids to enhance the home environment or foster social inclusion, benefits people with dementia as well as their partner by improving their quality of life, physical functions, psychosocial health and relationship satisfaction [15, 16]. Moreover, studies have indicated that hearing aid use confers a mitigating effect on the trajectories of cognitive decline by maintaining cognitive function, such as episodic memory [17, 18].

Although the mechanisms underlying the association of hearing loss with cognitive decline and dementia remain unclear, several hypotheses exist [19]. One possible mechanism is the shared pathologic etiology. The common factor theory suggested that the association of sensory and cognitive performance is explained by a third common factor, such as an aging brain [20, 21] or frailty syndrome [22]. The second hypothesis is biologically plausible given the effects of hearing loss on cognitive load and cognitive reserve, which may be mediated by social isolation or loneliness [12, 23]. One possible explanation for the association between social isolation, loneliness, and cognitive function is that loneliness or social isolation is associated with unhealthy behaviors or a plethora of chronic diseases related to poor cognition [24]. The third hypothesis is the information-degradation hypothesis, which posits that increased cognitive load from the compensation of auditory deficits may limit the resources available in performing other cognitive tasks [25].

One potential way in modifying the association of hearing loss with cognitive decline and dementia may be through increased participation in activities. Previous studies indicated that older adults with hearing loss were more likely to have smaller social networks [26]. Since the relationship between hearing loss and cognitive impairment may be mediated by social isolation or loneliness [24], efforts to improve the social networks of hearing-impaired older adults may be beneficial in preventing cognitive decline. Participation in leisurely activities, i.e. gardening, reading or engaging in hobbies has shown positive effects in reducing loneliness and feelings of isolation among older adults via social interaction and constructively spending time [27]. In addition to psychosocial benefits, participating in mentally stimulated leisurely activities may promote stability or enhance cognitive performance. A recent study found that poorer hearing function may affect verbal memory performance and attention to auditory stimuli among older adults [28], while another study focusing on the association between leisurely activity and cognitive function in old age indicated that more participation in self-improvement, intellectual, or cultural activities was associated with better performance in verbal ability and memory [29]. Maintaining a moderate or high level of participation in leisurely activities may play a role in the relationship between hearing loss and cognition. Engagement in leisurely activities makes up a considerable amount of daily activity among the Chinese elderly. Leisure activities may be demographically or culturally specific, and studies from Asian countries that have adopted Confucian culture suggested that the link between participating in leisure activities and cognitive performance may differ by sex [30, 31], as the elderly may possess differences in lifestyle and social networks. Additionally, consequences resulting from hearing loss, as well as the association between specific leisure activity and cognition, may also vary by sex. For example, older men with hearing loss were more likely to be depressed than men with normal hearing, while hearing loss did not affect the odds of depression in older women [32]. In contrast, elderly women with hearing loss tend to feel socially isolated, however, the association between hearing loss and social isolation was not significant among men [33]. Cognitively stimulating and socially engaging activities, along with intellectual or cultural activities, were found to reduce the risks of dementia among elderly men and women [29].

Though much research has documented that hearing loss was associated with cognitive decline and dementia in developed countries, a very limited number of studies have been done in developing countries, and few have focused on the role of engaging in leisurely activity. Maharani et al. [24] and Frank Lin et al. [12] put forward that the association between hearing loss and cognitive decline among the elderly may be mediated by cognitive load and/or social isolation. However, whether efforts in maintaining engagement in leisurely activities or increasing the size of social networks may mitigate the impact of hearing loss on cognition are still unclear. Given the expected rise of the aging population in China, a better understanding of such relationships would provide valuable insights into potential approaches in preventing or delaying the onset of dementia. According to the “cognitive load theory” and “cascade hypothesis” [34], this study aimed to examine: 1) the association of self-perceived hearing loss with cognitive function; 2) the role of engaging in leisurely activities as a potential moderator in this association; and 3) the differences in these associations by sex among the Chinese elderly.

## Methods

### Data source

We obtained the data from the Chinese Longitudinal Healthy Longevity Survey (CLHLS), a dynamic cohort study that collected first-wave data in 1998, along with six follow-up surveys with the replacement of deceased elders in 2000, 2002, 2005, 2008, 2011 and 2014 [35]. The CLHLS recruited a representative sample from 23 of the 31 provinces within China [36], and in-person interviews were conducted by the researchers so as to acquire data regarding demographics, socioeconomic status, lifestyle, and health. Consent for participation was given via face-to-face interviews, where informed consent was presented to the selected participants prior to the in-person interview. The informed consent included information on the interview’s duration, contents, and purpose, and the elderly participants were asked if they agreed to participate in the interview and be involved in a physical examination. If the participant declined to take the interview, the participate was allowed to ask his/her spouse, children or other relatives to answer on his/her behalf. The participant signed the informed consent if he/she was capable to read and write, otherwise, the informed consent was signed by participant’s consenting proxy. The relationship between the agent and the participant was included in the informed consent [35]. Details on the study design, sampling, measures, and data quality of the CLHLS are available elsewhere [37].

**Fig. 1:**
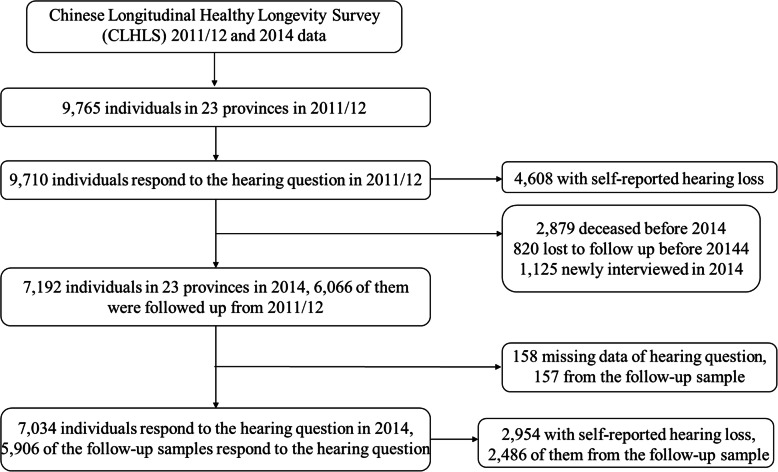
flowchart of the study sample

CLHLS included questions on self-reported hearing difficulties only in waves during 2011/12 and 2014. Therefore, the current study adopted two waves from the CLHLS longitudinal data taken during 2011/2012 to 2014. Accordingly, 9765 and 7192 respondents were interviewed in 2011/12 and 2014, respectively. Of the 9765 respondents interviewed in the 2011/12 survey, 6066 were followed up in the 2014 survey, but 820 respondents were lost to follow-up, while 2879 persons died or were already deceased prior to the 2014 follow-up survey. In addition to the 6066 follow-up respondents, 1125 respondents were newly interviewed in 2014 [38, 39]. Details concerning sample selection are presented in Fig. 1.

We restricted the study sample to 14,309 respondents aged ≥65 years who completed the questionnaire on self-reported hearing difficulties and cognitive function from the 2011/12–14 CLHLS sample. Here, 8844 were from the 2011/12 wave, while 5465 were part of the 2014 wave. Individuals were excluded if they were younger than 65 years of age, had died, or failed to respond to the items comprising the outcome variable.

### Measures

#### Self-reported hearing status

Hearing impairment was defined by responses of self-reported hearing difficulty. Participants were asked the following question to check their hearing status (without using hearing aids): “Do you have any difficulty with your hearing?” “YES” was coded as having hearing impairment, while “No” signified not having hearing impairment.

### Global cognition

Global cognition is measured using the Chinese version of the Mini-Mental State Examination (MMSE). The Chinese version of MMSE is comprised of 23 items and examines four aspects of cognitive function (orientation, calculation, recall, and language), which was adapted from the original MMSE developed by Folstein et al. [40]. Considering the cultural and socioeconomic conditions among the Chinse elderly, several items were modified or deleted to make the questions easily understandable and answerable among the participants whose cognitive function was normal. For example, the respondents were asked to name as many foods as possible rather than writing a complete sentence. The questions involving calculations were simplified, and one item pertaining to orientation to time and four items concerning orientation to place were excluded [41]. Several similar versions of the Chinese MMSE, which were all adopted according to Folstein’s version [40], have proven to be reliable and may be employed to study the Chinese elderly [42, 43]. Scores on each item are normally binary (e.g. 1 for a correct and 0 for otherwise). The total possible score on the MMSE is 30, with higher scores indicating better cognitive performance. In accordance with previous studies in China that suggested the use of 18 or 19 as the cutoff score of MMSE when screening for dementia among populations with little or no formal education [43, 44], individuals who scored < 18 were considered to be cognitively impaired. Hence, the 23-item used for the MMSE for Chinese elderly has demonstrated good validity [45].

### Leisure activity engagement

We used the frequency of participation in leisure activities to measure the level of engagement in such activities among older adults. Overall, the survey respondents were asked, “Do you now perform the following activities regularly? (please choose one from the frequency on the right)”. Each specific activity was indexed at five levels: 0 = never; 1 = not every month; 2 = not every week; 3 = not every day; and 4 = almost every day. To obtain a general perspective on activity involvement, the total sum of the scores regarding participation frequency in each activity was computed. Details concerning the leisure activities are shown in Additional file 1.

### Covariates

Covariates included several sociodemographic characteristics, health behaviors and health conditions that were potentially associated with cognitive function as in previous studies [46]. These variables consisted of age (continuous), sex (male vs. female), residence (urban vs. rural), education (illiterate vs. literate), smoking (never smoking, ever smoking vs. current smoking), alcohol use (never drinking, ever drinking vs. current drinking), self-reported general health status (good vs fair/poor), having been diagnosed with hypertension (yes vs. no), and having been diagnosed with diabetes (yes vs. no).

### Statistical analyses

Descriptive statistics were used for all study variables. Fixed effect models were used to capture the within-individual association between the occurrence of hearing loss and cognitive function decline over time. The fixed effect models are longitudinal models that were applied in the panel data, which confer benefits in treating unobserved confounders and time-invariant variables as a set of fixed parameters to control effects pertaining to both observed and unobserved time-invariant variables as well as the effect of years on the dependent variable [47]. In this study, Hausman tests were conducted, where the fixed effects model was shown to be more appropriate than the random effects model with chi2 = 49.13 (*p* < 0.005). Likelihood-ratio tests were also performed, which indicated that the fixed-effects model was more appropriate than the mixed-effects model with LR chi2 = − 3871.60 (*p* = 1.00). Moreover, the cognitive function was initially treated as a binary variable using a cutoff point of 18 (cognitively impaired vs. normal cognition). Fixed effect logit models were conducted to estimate the average odds ratios of cognitive decline related to the occurrence of self-perceived hearing loss during the follow-up period. The time-variant variables were adjusted in these models, including age, smoking, alcohol use, self-reported health, hypertension and diabetes. Second, the continuous form of the cognitive function (the summed MMSE scores) was utilized as the dependent variable to estimate the average change in MMSE scores associated with hearing loss during the follow-up period. Hausman tests indicated that fixed effects models were more appropriate in the estimation compared to random effects models (chi2 = 167.06, *p* < 0.001). Furthermore, regarding the potential bias caused by the missing data due to participants being deceased or lost to follow-up, the characteristics of the missing data were reported, and a proportional hazard model was done for the mortality sample as well as a regression analysis using an attribution indicator and baseline dataset, as shown in the Supplementary document. All analyses were conducted using the Stata 14.0 software.

## Results

### Sample characteristics

Table 1 presents the sample characteristics over two waves of CLHLS. The mean MMSE scores slightly increased from 26.08 (SD = 5.35) in wave 1 to 26.51 (SD = 4.67) in wave 2. The proportion of participants having cognitive impairment decreased from 8% (*n* = 749) to 6% (*n* = 336) over two waves, while the proportion for hearing loss decreased from 44% (*n* = 3937) in wave 1 to 39% (*n* = 2137) in wave 2. The mean scores for all kinds of leisurely activities increased over time from 9.98 to 10.48 over two waves. The average age was 85.08 (SD = 10.98) in wave 1 and 84.74 (SD = 9.99) in wave 2. Details regarding the sample characteristics are shown in Table 1.

**Table 1:** Characteristics of participants by waves in CLHLS, 2011/2012 to 2014, mean (SD)/n (%)

Variables	Wave 1			Wave 2		
	Total (*n* = 8844)	Male (*n* = 4089)	Female (*n* = 4755)	Total (*n* = 5465)	Male (*n* = 2628)	Female (2837)
MMSE scores^a^	26.08(5.35)	27.13(4.44)	25.19(5.88)	26.51(4.67)	27.46(3.88)	25.66(4.47)
Cognitive impairment^b^	749(8.47)	189(4.62)	560(11.78)	336(6.19)	97(3.69)	239(8.42)
Self-reported HL^b^	3937(44.52)	1706(41.72)	2231(46.92)	2137(39.38)	997(37.94)	1141(40.22)
Activity engagement^a^	9.98(6.62)	11.43(6.58)	8.73(6.39)	10.48(6.65)	11.61(6.64)	9.51(6.54)
Productive activities^a^	2.13(1.90)	2.04(1.88)	2.21(1.91)	2.27(1.88)	2.11(1.87)	2.43(1.88)
Outdoor activities^a^	2.21(1.84)	2.45(1.79)	2.01(1.85)	2.22(1.84)	2.39(1.81)	2.07(1.85)
Intellectual activities^a^	0.57(0.98)	0.87(1.13)	0.31(0.75)	0.61(1.02)	0.90(1.15)	0.35(0.80)
Recreational activities^a^	1.40(1.10)	1.61(1.07)	1.22(1.10)	1.49(1.09)	1.65(1.06)	1.35(1.10)
Social activities^a^	0.30(0.87)	0.38(0.96)	0.24(0.78)	0.32(0.89)	0.39(0.96)	0.27(0.83)
Age^a^	85.08(10.98)	82.84(10.06)	87.01(11.37)	84.74(9.99)	82.97(9.16)	86.13(10.63)
Rural^b^	4624(52.28)	2097(51.28)	2527(53.14)	2835(52.83)	1366(52.46)	1501(53.63)
Illiterate^b^	4987(56.39)	1294(31.65)	3693(77.67)	2825(52.05)	771(29.34)	2066(72.82)
Ever smoking^b^	1439(16.36)	1158(28.47)	281(5.94)	774(14.33)	654(24.99)	123(4.36)
Current smoking^b^	1639(18.64)	1370(33.68)	269(5.69)	977(18.09)	843(32.21)	144(5.11)
Ever drinking^b^	1270(14.51)	947(23.43)	323(6.86)	575(10.68)	463(17.72)	114(4.06)
Current drinking^b^	1520(17.37)	1142(28.26)	378(8.03)	876(16.28)	716(27.40)	167(5.95)
Self-reported poor health^b^	1501(17.22)	581(14.37)	920(19.69)	856(15.97)	366(14.04)	492(17.62)
Hypertension^b^	3126(50.76)	1388(49.62)	1738(51.71)	2167(56.97)	1011(55.19)	1168(58.43)
Diabetes^b^	1118(21.73)	528(22.34)	590(21.22)	904(28.99)	437(28.52)	476(29.55)

^a^ variable being reported with mean (SD). ^b^ variable being reported with number (proportion)

Elderly females had a higher MMSE score than males in both waves. The proportion of cognitive impairment and self-reported hearing loss was found to be higher among older females than in males, with 12% (*n* = 560) of females having cognitive impairment (vs. 5% for male) and 47% (*n* = 2231) of females having hearing loss (vs. 42% for male) in wave 1. In these two waves, the elderly male cohort were reported to frequently engage in leisurely activities compared to females. Men were observed to have higher average scores in physical outdoor activities, intellectual activities, recreational activities, and social activities, while women participated more in productive activities. Older men attained better education than older women, with 77% (*n* = 3693) of females being illiterate (vs. 32% male) in wave 1 and 73% (vs. 29% male) in wave 2, respectively. Details regarding the characteristics of the elderly sex subgroups are presented in Table 1. With respect to the sample that was excluded due to participants’ death or loss to follow-up, elderly participants among the excluded sample were more likely to have a higher mean age (91.3 years old), be hearing impaired, illiterate and have poorer self-reported health (see Supplementary).

### Association of hearing impairment and cognitive impairment

Table 2 illustrates the results of the association between the co-occurrence of self-reported hearing loss and cognitive impairment during the follow-up period using fixed-effect models. The unadjusted model (Model 1) shows that hearing loss was significantly associated with the occurrence of cognitive impairment. Compared to participants without hearing loss, those who reported hearing loss during the follow-up period were 1.87 times more likely to develop cognitive impairment (odds ratio [OR] = 1.87, 95% confidence intervals [CI] = 1.28, 2.74). After adjusting with the time-variant covariates in Model 2, the association between hearing loss and cognitive impairment was consistent, with OR = 2.48 (95%CI:1.22,5.06). An interaction between sex and hearing loss is introduced in Model 3, which demonstrates that the association of hearing loss and cognitive impairment did not differ by sex. In Model 4, we employed an interactive term of hearing loss and leisurely activity participation to test whether participants who frequently participated in leisurely activities were found to have a mitigated association of hearing loss and cognitive impairment. However, the results show that those who actively participated in leisurely activities were not found to have an association between hearing loss and the occurrence of cognitive impairment.

**Table 2:** Odds ratio and 95% confidence interval for fixed effects models on the association between hearing loss and cognitive decline during 2011 to 2014

Variables	Model 1	Model 2	Model 3	Model 4
**Self-reported HL**	1.87(1.28,2.74)	2.48(1.22,5.06)	2.90(0.75,11.29)	2.84(1.00,8.13)
Ever smoking		0.54(0.08,3.68)	0.57(0.08,4.09)	0.50(0.07,3.46)
Current smoking		0.45(0.08,2.49)	0.45(0.08,2.49)	0.41(0.07,2.26)
Ever drinking		4.56(0.92,22.60)	4.44(0.89,22.20)	3.96(0.78,20.13)
Current drinking		0.21(0.05,0.89)	0.21(0.05,0.88)	0.18(0.04,0.82)
Poor self-reported health		2.17(0.93,5.08)	2.15(0.92,5.04)	2.09(0.88,5.01)
Hypertension		0.53(0.25,1.09)	0.52(0.25,1.08)	0.51(0.24,1.07)
Diabetes		0.77(0.34,1.75)	0.77(0.34,1.77)	0.78(0.34,1.78)
Self-reported HI * female			0.80(0.16,3.92)	
Self-reported HI * activity engagement				0.98(0.88,1.09)
Year	1.69(1.38,2.07)	1.91(1.26,2.90)	1.90(1.25,2.89)	1.78(1.15,2.74)
**Observation**	830	266	266	266

The analysis regarding the association of self-reported hearing loss and change in global cognition during the follow-up period was further elucidated using linear fixed-effect models, which are presented in Table 3. After adjusting for time-variant covariates, participants who reported hearing loss during the follow-up period suffered from a 0.81-point decrease in cognition score. This association remains significant in both sex subgroups, with a 0.79-point decrease in cognition scores among males and a 0.85-point decrease among females. An interactive term of hearing loss and leisurely activity participation was then used in the adjusted models, which signified that frequently engaging in leisurely activities conferred a moderative role in the association between hearing loss and cognitive function. Accordingly, Table 3 shows that among those with normal hearing in the baseline as well as those who reported hearing loss in the follow-up periods, individuals who frequently took part in leisurely activities had a less likelihood of cognitive decline. Similar results were found within the male subgroup, however, the female subgroup demonstrated that the interactions of hearing loss on leisurely activities were not significantly associated with cognitive function.

**Table 3:** Estimated coefficients and 95% confidence interval for fixed effects models on the association of hearing loss and MMSE scores, 2011/12–2014

Variables	Adjusted models			Adjusted models + activity engagement			Adjusted models+ interaction for HL and activity engagement		
	Total	Male	Female	Total	Male	Female	Total	Male	Female
**Self-reported HL**	−0.81(−1.22, −0.39)	−0.79(−1.31,-0.27)	−0.85(−1.49,-0.21)	−0.82(−1.23, −0.41)	−0.82(−1.33, −0.30)	−0.84(−1.47, −0.21)	−1.45(−2.17,-0.73)	−1.85(−2.80,-0.89)	−1.15(−2.23,-0.08)
Activity engagement				0.09(0.06, 0.13)	0.08(0.04,0.12)	0.11(0.05,0.17)	0.07(0.03–0.11)	0.05(−0.001,0.09)	0.10(0.03,0.17)
**Interaction**									
Activity engagement*HL							0.06(0.003,0.11)	0.09(0.02,0.16)	0.03(−0.05,0.12)
Covariates	Yes	Yes	Yes	Yes	Yes	Yes	Yes	Yes	Yes
**Constant**	26.77	27.61	26.04	25.75	26.62	24.95	26.04	27.03	25.10
**Observations**	7942	3735	4207	7942	3735	4207	7942	3735	4207

## Discussion

This study investigated the association between hearing impairment and cognitive function in a nationwide population-based survey on the Chinese elderly. After identifying the impacts of hearing deprivation on cognitive decline, whether engaging in leisurely activities moderated the link between hearing loss and cognitive function from the perspective of gender was explored. To the best of our knowledge, this is the first study to report the empirical results of hearing loss in relation to cognitive function as well as the moderative role of leisurely activities from a longitudinal survey in mainland China. It was found that elderly males or females who reported as having a self-perceived hearing difficulty had a greater risk of cognitive impairment. Frequent participation in leisurely activities benefited older adults with hearing loss to perform better in cognitive functioning, particularly in the male subgroup.

The results of this study contribute to studies exploring the association between hearing impairment and cognitive function among the aging Chinese population. Our findings align well with those of previous studies [12, 46, 48], documenting a significant correlation between hearing difficulty and poorer cognitive performance among the elderly. In contrast, other studies have yielded inconsistent findings [49, 50]. Poorer hearing function may be related to certain domains of cognitive functional decline, especially concerning memory and executive function [51]. The variability in assessing cognitive function and how one may define hearing loss may result in conflicting findings [11]. In addition, past studies were conducted in non-representative populations, which may give rise to sample selection bias, leading to inaccurate results.

Previous studies suggested that compared to men, women are more easily affected by the risk factors of cognitive impairment, as women have more rapid declines in hearing sensitivity at certain ranges of frequency [52], higher CVD (cardiovascular diseases) risks [53] and an increased likelihood social isolation [28]. However, our results do not imply sex difference in the association between hearing loss and cognitive impairment.

In the current study, frequent engagement in leisure activities played a moderating role in the association between hearing loss and cognitive decline. Previous studies focusing on the relationship of hearing loss and cognition considered activities engagement or social isolation as a mediator [23], or only tested the moderator role of hearing aids, length of time with treatment and age [54], seldom regarding the activity engagement on the association between hearing health and cognition. Communication breakdown caused by hearing loss may affect the types and frequency of leisure activities that older adults participate in. Hearing-impaired older adults may have to cope with verbal challenges and anxiety stress in the presence of social gatherings. Therefore, performance of auditory function may affect older adults’ engagement in some social activities and hearing-related recreational activities (e.g. listening to music and watching television), which have been proved with cognitive benefits [55, 56]. Moreover, hearing loss may give rise to basic and instrumental activities of daily living loss [57]. Engagement in productive activities, such as caregiving or doing housework, may require hearing-impaired older adults more cognitive capacity to deal with complex cognitive tasks. Leisure activities involve physical, mental, and social components [58]. Compared to productive activities, engagement in leisure activities, such as personal slow walking or outdoor exercising, may require less cognitive loads or social interaction burden. Active participated in leisure activities, such as reading or knitting, can benefit hearing-impaired older adults’ cognition by fostering intellectual stimulation, mood improvement that are related to cognitive maintenance [59].

Sex difference is shown in the moderating effect of leisure activities between hearing loss and cognitive decline. Although frequent engaged in leisure activities mitigated the effects of hearing loss on cognitive decline among older males, we did not find any statistically significant association between the interaction of various types of leisure activities on hearing loss and cognition among the female sample. The moderating effect of leisure activities is more pronounced among older men than women may be because women in China tend to have lower education and less likely to accumulate socioeconomic resources [60], thus resulting in fewer cognitive resources and less benefit from intelligence stimulating activities, such as reading books or magazines. Additionally, older women are generally more likely to sedentary or less active in leisure-time physical activity than men. Older women may perceive more barriers to outdoor activities than men, especially in a condition of perceived poor health status [61].

The current study has several important limitations. First, our measurement of hearing loss is based on a dichotomized measure of self-reported hearing loss and verbal cognitive test, which may limit the accuracy of our estimations among older adults due to measurement bias. Although Kiely’s et al. (2012) study documented a moderate association between self-reported and audiometric hearing loss and suggested that self-reported hearing loss may indicate hearing disability, the dichotomized measure of self-reported hearing loss does not provide a very accurate and reliable basis to some extent compared with audiometric hearing loss [62]. Moreover, self-reported items may also be biased by correlated measurement error or same-source bias, such as age, sex or cognitive function. Third, we do not have data on the duration of hearing loss and severity of hearing loss, thus we are not able to identify the exact impact of hearing loss on cognitive function. Further studies are needed with audiometric measures and sufficient information on hearing function to understand the association of hearing loss and cognitive function. The relatively short follow-up period for self-reported hearing loss and cognition contributes to another potential limitation by increasing the uncertainty of our estimates. A further limitation to our main results may come from the potential bias related to sample attrition due to mortality and loss to follow-up. Approximately 38% of the sample from the wave 2011/12 died or lost to follow-up in wave 2014, which may result in significant bias in our estimations. Although our analysis for missing data suggested that the association between hearing loss and cognitive decline was not differed systematically by the follow-up sample or those known to be deceased (see Supplementary), some caution is still needed in interpreting these results regarding the limitations mentioned above.

Despite these limitations, strengths of our current are that our results are based on a nationally representative sample, and a longitudinal analysis with fixed-effect model methods to avoid the potentially strong cross-sectional confounding effects and fix problems on time-invariant omitted variables to some extent. If our results are confirmed by a standard audiometric testing protocol and in other independent studies, our findings potentially have important implications in aging health. Our findings show that hearing loss was negatively associated with cognitive decline and of importance to highlight the role of leisure activities engagement in moderating the association. This implies the Chinese policy maker to consider the role of hearing aids and activity participation in cognition or dementia prevention program.

## Conclusions

Hearing impairment was negatively associated with cognitive function among older adults in China. Leisure activity moderated the impact of hearing loss on cognitive performance among older men rather than women. These findings lend support to the hypothesis that hearing impairment may be a risk factor for cognitive dysfunction in older adults and that hearing aid use or proper leisure activity engagement could possibly reduce this risk. Given the current lack of standardized audiometric assessment and mechanism identification, further studies are needed to estimate whether hearing loss and leisure activity interventions could reduce cognitive decline in older adults.

## Supplementary information

**Additional file 1: Table 1.** Classification of leisure activities. **Table 2.** Characteristics at baseline for missing sample (deceased or lost to follow up). **Table 3.** Cox proportional hazard model for mortality sample. **Table 4.** Regression for cognitive impairment with an indicator for attrition using sample from 2011/12 wave.

## Data Availability

The data that support the findings of this study are available from Chinese Longitudinal Healthy Longevity Survey (CLHLS) but restrictions apply to the availability of these data, which were used under license for the current study, and so are not publicly available. Data are however available from the authors upon reasonable request and with permission of Chinese Longitudinal Healthy Longevity Survey (CLHLS).

## Abbreviations

CLHLS: China Longitudinal Healthy Longevity Survey
MMSE: Modified Mini Mental Examination
MCI: Mild cognitive impairment

## References

[1] World Health Organization. Dementia. https://www.who.int/en/news-room/fact-sheets/detail/dementia. Accessed 20 Sept 2019.

[2] National Bureau of Statistics P. 2010 sixth national population census key data bulletin. http://www.stats.gov.cn/tjsj/tjgb/rkpcgb/qgrkpcgb/201104/t20110428_30327.html. Accessed 20 Sept 2019.

[3] Wu YT, Lee H, Norton S, Chen C, Chen H, He C, et al. Prevalence studies of dementia in mainland China, Hong Kong and Taiwan: a systematic review and meta-analysis. PLoS One. 2013. 10.1371/journal.pone.0066252. 10.1371/journal.pone.0066252 PMC3679068 23776645

[4] Xu J, Wang J, Wimo A, Fratiglioni L, Qiu C. The economic burden of dementia in China, 1990–2030: implications for health policy. Bull World Health Organ. 2016. 10.2471/blt.15.167726. 10.2471/BLT.15.167726 PMC5180346 28053361

[5] Petersen RC, Doody R, Kurz A, et al. Current concepts in mild cognitive impairment[J]. Arch Neurol. 2001;58(12):1985–92. 10.1001/archneur.58.12.1985 11735772

[6] Nie HW, Xu Y, Liu B, Zhang YD, Lei T, Hui XP (2011). The prevalence of mild cognitive impairment about elderly population in China: a meta-analysis. Int J Geriatr Psychiatry.

[7] World Health Organization. World report on ageing and health. https://www.who.int/ageing/events/world-report-2015-launch/en/. Accessed 22 Sept 2019.

[8] Newson RS, Kemps EB (2005). General lifestyle activities as a predictor of current cognition and cognitive change in older adults: a cross-sectional and longitudinal examination. J Gerontol Ser B Psychol Sci Soc Sci.

[9] Plassman BL, Williams JW, Burke JR, Holsinger T, Benjamin S (2010). Systematic review: factors associated with risk for and possible prevention of cognitive decline in later life. Ann Intern Med.

[10] Lin FR, Metter EJ, O’brien RJ, Resnick SM, Zonderman AB, Ferrucci L (2011). Hearing loss and incident dementia. Arch Neurol.

[11] Loughrey DG, Kelly ME, Kelley GA, Brennan S, Lawlor BA (2018). Association of age-related hearing loss with cognitive function, cognitive impairment, and dementia: a systematic review and meta-analysis. JAMA Otolaryngol Head Neck Surg.

[12] Lin FR, Yaffe K, Xia J, Xue QL, Harris TB, Purchase HE (2013). Hearing loss and cognitive decline in older adults. JAMA Intern Med.

[13] Yu L, Sun X, Wei Z, Wang Q, Qu CY (2008). A study on the status quo of aged population with hearing loss in China. Chin Sci J Hear Speech Rehabil.

[14] Ford AH, Hankey GJ, Yeap BB, Golledge J, Flicker L, Almeida OP (2018). Hearing loss and the risk of dementia in later life. Maturitas.

[15] Hooper E, Simkin Z, Abrams H (2019). Feasibility of an intervention to support hearing and vision in dementia: the SENSE-Cog Field Trial. J Am Geriatr Soc.

[16] Leroi I, Simkin Z, Hooper E, et al. Impact of an intervention to support hearing and vision in dementia: the SENSE-Cog Field Trial[J]. Int J Geriatr Psychiatry. 2020;35(4):348–57. 10.1002/gps.5231 PMC7079053 31713262

[17] Amieva H, Ouvrard C, Giulioli C, Meillon C, Rullier L, Dartigues JF (2015). Self-reported hearing loss, hearing aids, and cognitive decline in elderly adults: a 25-year study. J Am Geriatr Soc.

[18] Maharani A, Dawes P, Nazroo J (2018). Longitudinal relationship between hearing aid use and cognitive function in older Americans. J Am Geriatr Soc.

[19] Wayne RV, Johnsrude IS (2015). A review of causal mechanisms underlying the link between age-related hearing loss and cognitive decline. Ageing Res Rev.

[20] Anstey KJ, Luszcz MA, Sanchez L (2001). A reevaluation of the common factor theory of shared variance among age, sensory function, and cognitive function in older adults. J Gerontol Ser B Psychol Sci Soc Sci.

[21] Lindenberger U, Baltes PB (1994). Sensory functioning and intelligence in old age: a strong connection. Psychol Aging.

[22] Panza F, Solfrizzi V, Seripa D, Imbimbo BP, Capozzo R, Quaranta N (2015). Age-related hearing impairment and frailty in Alzheimer’s disease: interconnected associations and mechanisms. Front Aging Neurosci.

[23] Dawes P, Emsley R, Cruickshanks KJ, Imbimbo BP, Capozzo R, Quaranta N (2015). Hearing loss and cognition: the role of hearing AIDS, social isolation and depression. PLoS One.

[24] Maharani A, Pendleton N, Leroi I (2019). Hearing impairment, loneliness, social isolation, and cognitive function: longitudinal analysis using English longitudinal study on ageing. Am J Geriatr Psychiatry.

[25] Lautenschlager NT, Cox KL, Flicker L, Foster JK, van Bockxmeer FM, Xiao J (2008). Effect of physical activity on cognitive function in older adults at risk for Alzheimer disease: a randomized trial. JAMA.

[26] Ogawa T, Uchida Y, Nishita Y (2019). Hearing-impaired elderly people have smaller social networks: a population-based aging study. Arch Gerontol Geriatr.

[27] Gardiner C, Geldenhuys G, Gott M (2018). Interventions to reduce social isolation and loneliness among older people: an integrative review. Health Soc Care Community.

[28] Armstrong NM, An Y, Ferrucci L (2020). Temporal sequence of hearing impairment and cognition in the Baltimore longitudinal study of aging. J Gerontol A Biol Sci Med Sci.

[29] Hassing LB (2020). Gender differences in the association between leisure activity in adulthood and cognitive function in old age: a prospective longitudinal population-based study. J Gerontol B Psychol Sci Soc Sci.

[30] Lee Y, Yeung WJJ. Gender matters: productive social engagement and the subsequent cognitive changes among older adults[J]. Soc Sci Med. 2019;229:87–95. 10.1016/j.socscimed.2018.08.024 30177360

[31] Feng L, Ng XT, Yap P, Li J, Lee TS, Håkansson K (2014). Marital status and cognitive impairment among community-dwelling Chinese older adults: the role of gender and social engagement. Dement Geriatr Cogn Dis Extra.

[32] Harada S, Nishiwaki Y, Michikawa T (2008). Gender difference in the relationships between vision and hearing impairments and negative well-being. Prev Med.

[33] Uchida Y, Sugiura S, Nishita Y (2019). Age-related hearing loss and cognitive decline—the potential mechanisms linking the two. Auris Nasus Larynx.

[34] Mick P, Kawachi I, Lin FR (2014). The association between hearing loss and social isolation in older adults. Otolaryngol Head Neck Surg.

[35] Gu D. General data quality assessment of the CLHLS. Healthy longevity in China. Dordrecht: Springer; 2008. p. 39–60.

[36] Gu D, Dupre ME. Assessment of reliability of mortality and morbidity in the 1998–2002 CLHLS waves. Healthy longevity in China. Dordrecht: Springer; 2008. p. 99–116.

[37] Zeng Y. Introduction to the chinese longitudinal healthy longevity survey (CLHLS). Healthy longevity in China. Dordrecht: Springer; 2008. p. 23–38.

[38] Zeng Y (2016). Chinese Longitudinal Healthy Longevity Survey (CLHLS), 1998-2014.

[39] Zeng Y (2016). Chinese Longitudinal Healthy Longevity Survey (CLHLS), 2014.

[40] Folstein MF, Folstein SE, McHugh PR (1975). “Mini-mental state”: a practical method for grading the cognitive state of patients for the clinician. J Psychiatr Res.

[41] Zeng Y, Vaupel JW (2002). Functional capacity and self–evaluation of health and life of oldest old in China. J Soc Issues.

[42] Yu ES, Liu WT, Levy P, Zhang M, Katzman R, Lung C (1989). Cognitive impairment among elderly adults in Shanghai, China. J Gerontol.

[43] Zhang Z, Gu D, Hayward MD (2008). Early life influences on cognitive impairment among oldest old Chinese. J Gerontol Ser B Psychol Sci Soc Sci.

[44] Zhang ZX, Zahner GE, Roman GC, Liu XH, Wu CB, Hong Z (2006). Socio-demographic variation of dementia subtypes in China: methodology and results of a prevalence study in Beijing, Chengdu, Shanghai, and Xian. Neuroepidemiology.

[45] Zhong BL, Chen SL, Tu X, Conwell Y (2017). Loneliness and cognitive function in older adults: findings from the chinese longitudinal healthy longevity survey. J Gerontol B Psychol Sci Soc Sci.

[46] Lin FR (2011). Hearing loss and cognition among older adults in the United States. J Gerontol A Biomed Sci Med Sci.

[47] Allison PD. Fixed effects regression models. California: SAGE publications; 2009.

[48] Sugawara N, Sasaki A, Yasui-Furukori N, Kakehata S, Umeda T, Namba A (2011). Hearing impairment and cognitive function among a community-dwelling population in Japan. Ann General Psychiatry.

[49] Lin MY, Gutierrez PR, Stone KL, Yaffe K, Ensrud KE, Fink HA (2004). Vision impairment and combined vision and hearing impairment predict cognitive and functional decline in older women. J Am Geriatr Soc.

[50] Bucks RS, Dunlop PD, Taljaard DS (2016). Hearing loss and cognition in the Busselton Baby Boomer cohort: an epidemiological study. Laryngoscope.

[51] Lin FR, Ferrucci L, Metter EJ, Brennan-Jones CG, Hunter M, Wesnes K, Eikelboom RH (2011). Hearing loss and cognition in the Baltimore longitudinal study of aging. Neuropsychology.

[52] Wiley TL, Chappell R, Carmichael L, Nondahl DM, Cruickshanks KJ (2008). Changes in hearing thresholds over 10 years in older adults. J Am Acad Audiol.

[53] Helzner EP, Patel AS, Pratt S, Sutton TK, Cauley JA, Talbott E (2011). Hearing sensitivity in older adults: associations with cardiovascular risk factors in the health, aging and body composition study. J Am Geriatr Soc.

[54] Taljaard DS, Olaithe M, Brennan-Jones CG, Eikelboom RH, Bucks RS (2016). The relationship between hearing impairment and cognitive function: a meta - analysis in adults. Clin Otolaryngol.

[55] Särkämö T, Tervaniemi M, Laitinen S, Numminen A, Kurki M, Johnson JK, Rantanen P (2014). Cognitive, emotional, and social benefits of regular musical activities in early dementia: randomized controlled study. The Gerontologist.

[56] Kawachi I, Berkman LF (2001). Social ties and mental health. J Urban Health.

[57] Yamada M, Nishiwaki Y, Michikawa T, Takebayashi T (2012). Self-reported hearing loss in older adults is associated with future decline in instrumental activities of daily living but not in social participation. J Am Geriatr Soc.

[58] Fratiglioni L, Paillard-Borg S, Winblad B (2004). An active and socially integrated lifestyle in late life might protect against dementia. Lancet Neurol.

[59] Ghisletta P, Bickel JF, Lövdén M (2006). Does activity engagement protect against cognitive decline in old age? Methodological and analytical considerations. J Gerontol Ser B Psychol Sci Soc Sci.

[60] Lei X, Hu Y, Mcardle JJ, Smith JP, Zhao Y (2012). Gender differences in cognition among older adults in China. J Hum Resour.

[61] Leern YS (2005). Gender differences in physical activity and walking among older adults. J Women Aging.

[62] Kiely KM, Gopinath B, Mitchell P (2012). Evaluating a dichotomized measure of self-reported hearing loss against gold standard audiometry: prevalence estimates and age bias in a pooled national data set. J Aging Health.

