# Supplementary material for: Hearing loss and cognitive function among Chinese older adults: the role of participation in leisure activities
Source: BMC Geriatr. 2020 Jun 19;20:215. doi: 10.1186/s12877-020-01615-7 (PMC7305626; doi:10.1186/s12877-020-01615-7)
Supplement: Supplementary file 1 — Additional file 1: Table 1. Classification of leisure activities. Table 2. Characteristics at baseline for missing sample (deceased or lost to follow up). Table 3. Cox proportional hazard model for mortality sample. Table 4. Regression for cognitive impairment with an indicator for attrition using sample from 2011/12 wave. [file 12877_2020_1615_MOESM1_ESM.docx]

Table 1 Classification of leisure activities

| Types | Items |
| --- | --- |
| Productive | Housework, such as cooking, taking care of kids |
| Physical | Personal outdoor activities, such as slow walking |
| Intellectual | Reading books/ newspapers |
|  | Play cards and/or mahjong |
| Recreational | Watch television and/or listen to radio |
|  | Garden work |
|  | Raise domestic animals |
| Social | Social activities (organized) |

Table 2 Characteristics at baseline for missing sample (deceased or lost to follow up)

| Characteristics | Mean (SD)/ n(%) |
| --- | --- |
| MMSE score | 24.41(6.58) |
| Cognitive impairment |  |
| Yes | 472(12.76) |
| No | 3227(87.24) |
| Self-reported hearing impairment |  |
| Yes | 2222(60.56) |
| No | 1447(39.44) |
| Domestic activities | 2.05(2.96) |
| Solitary leisure activities | 3.88(3.63) |
| Social activities | 0.47(1.26) |
| Age | 91.30(10.54) |
| Residence |  |
| Urban | 1779(48.09) |
| Rural | 1920(51.91) |
| Education |  |
| Illiterate | 2385(64.48) |
| Literate | 1314(35.52) |
| Smoking |  |
| Non-smoker | 2480(68.09) |
| Ex-smoker | 643(17.66) |
| Current smoker | 519(14.25) |
| Drinking |  |
| Non-drinker | 2555(70.56) |
| Ex-drinker | 564(15.58) |
| Current drinker | 502(13.86) |
| Self-reported health |  |
| Good/fair self-reported health | 2448(78.54) |
| Poor self-reported health | 669(21.46) |

Table 3. Cox proportional hazard model for mortality sample.

| Variables | Haz. Ratio | P | 95% CI |
| --- | --- | --- | --- |
| Hearing impairment | 1.07 | 0.193 | (0.97, 1.19) |
| Domestic activities | 0.71 | 0.000 | (0.67, 0.75) |
| Solitary leisure activities | 0.91 | 0.000 | (0.87, 0.95) |
| Social activities | 0.87 | 0.001 | (0.79, 0.95) |
| Age | 1.06 | 0.000 | (1.05, 1.06) |
| Smoker |  |  |  |
| Ex-smoker | 1.37 | 0.000 | (1.20, 1.56) |
| Current smoker | 1.04 | 0.810 | (0.76, 1.41) |
| Regular-smoker | 1.13 | 0.135 | (0.96, 1.32) |
| Drinker |  |  |  |
| Ex-drinker | 1.18 | 0.016 | (1.03, 1.36) |
| Current drinker | 1.00 | 0.994 | (0.80, 1.25) |
| Regular-drinker | 1.04 | 0.665 | (0.88, 1.23) |
| Self-reported health |  |  |  |
| Fair self-reported health | 1.16 | 0.005 | (1.05, 1.29) |
| Poor self-reported health | 1.34 | 0.000 | (1.18, 1.53) |
| Observation |  |  |  |

Table 4. Regression for cognitive impairment with an indicator for attrition using sample from 2011/12 wave.

| Variable | Cognitive |
| --- | --- |
| Hearing impairment | -1.163(0.111) *** |
| Sex | -0.810(0.128) *** |
| Domestic activities | 0.644(0.053) *** |
| Solitary leisure activities | 0.573(0.048) *** |
| Social activities | 0.243(0.070) *** |
| Age | -0.095(0.006) *** |
| Ex-smoker | 0.157(0.160) |
| Current smoker | 0.196(0.333) |
| Regular-smoker | -0.128(0.164) |
| Ex-drinker | -0.012(0.158) |
| Current drinker | -0.018(0.244) |
| Regular-drinker | 0.025(0.169) |
| Fair self-reported health | -0.333(0.112) ** |
| Poor self-reported health | -1.026(0.147) *** |
| Constant | 33.472(0.539) *** |
|  | 0.594(1.687) |
| Attrition*Hearing impairment | 0.081(0.397) |
| Attrition*Sex | 0.211(0.421) |
| Attrition*Domestic activities | -0.026(0.194) |
| Attrition*Solitary leisure activities | -0.287(0.165) |
| Attrition*Social activities | 0.030(0.224) |
| Attrition*Age | 0.001(0.018) |
| Attrition*Ex-smoker | 0.288(0.579) |
| Attrition*Current smoker | -2.590(1.195) * |
| Attrition*Regular-smoker | 0.385(0.554) |
| Attrition*Ex-drinker | -0.713(0.638) |
| Attrition*Current drinker | 0.265(0.929) |
| Attrition*Regular-drinker | -0.264(0.588) |
| Attrition*Fair self-reported health | 0.227(0.396) |
| Attrition*Poor self-reported health | -0.175(0.527) |
| Observations | 8,643 |
| R-squared | 0.234 |
| F-statistic for test on the joint effect of attrition on: |  |
| constant and coefficient estimates | F = 1.09, *P* = 0.360 |
| constant and coefficient estimates | F = 1.04, *P =* 0.410 |
| constant and coefficient estimates | F = 0.12, *P* = 0.725 |
